# E3 ligase TRIM25 ubiquitinates RIP3 to inhibit TNF induced cell necrosis

**DOI:** 10.1038/s41418-021-00790-3

**Published:** 2021-05-05

**Authors:** Pucheng Mei, Feiyan Xie, Jiasong Pan, Sen Wang, Wenqing Gao, Rui Ge, Baocai Gao, Siqi Gao, Xiangjun Chen, Yongming Wang, Jiaxue Wu, Chen Ding, Jixi Li

**Affiliations:** 1 State Key Laboratory of Genetic Engineering, Department of Neurology, School of Life Sciences and Huashan Hospital, MOE Engineering Research Center of Gene Technology, Shanghai Engineering Research Center of Industrial Microorganisms, Fudan University, Shanghai, 200438 China; 2 State Key Laboratory of Genetic Engineering, School of Life Sciences, Fudan University, Shanghai, 200438 China; 3 Department of Neurology, Huashan Hospital, Fudan University, Shanghai, 200040 China

**Keywords:** Cell death and immune response, Immune cell death

## Abstract

Receptor interacting protein kinase 3 (RIP3 or RIPK3), the critical executor of cell programmed necrosis, plays essential roles in maintaining immune responses and appropriate tissue homeostasis. Although the E3 ligases CHIP and PELI1 are reported to promote RIP3 degradation, however, how post-translational modification regulates RIP3 activity and stability is poorly understood. Here, we identify the tripartite motif protein TRIM25 as a negative regulator of RIP3-dependent necrosis. TRIM25 directly interacts with RIP3 through its SPRY domain and mediates the K48-linked polyubiquitination of RIP3 on residue K501. The RING domain of TRIM25 facilitates the polyubiquitination chain on RIP3, thereby promoting proteasomal degradation of RIP3. Also, TRIM25 deficiency inhibited the ubiquitination of RIP3, thus promoting TNF-induced cell necrosis. Our current finding reveals the regulating mechanism of polyubiquitination on RIP3, which might be a potential therapeutic target for the intervention of RIP3-dependent necrosis-related diseases.

## Introduction

Cell programmed necrosis (also called necroptosis) acts as an alternative way of apoptosis when apoptosis is inhibited, functioning in defense of pathogen-infected cells and damaged cells during inflammatory stimulations [1, 2]. Necroptosis can be triggered by multiple signaling pathways, including those initiated by death receptors (TNFR), TLRs, RLRs, and DNA virus sensor DAI [3, 4]. Receptor interacting protein kinase 3 (RIP3 or RIPK3) and its family protein RIP1 (or RIPK1) forms a large complex called necrosome, which is required in the TNF induced cell necroptosis [5–7]. The RIP1/RIP3 necrosome assemblies into a heterodimeric fibril and shows typical structural characterizations with β-amyloid [8–10]. Among the complex, RIP3 is a switch molecule for the interconversion between apoptosis and programmed cell necrosis [6]. Phosphorylated RIP3 binds with and phosphorylates its downstream protein mixed lineage kinase domain-like (MLKL), promoting MLKL oligomerization, eventually triggering necrotic cell death [5, 11–14].

Protein translation modifications, including phosphorylation, ubiquitination, and deubiquitination, play essential roles in the process of cell necrosis [5, 15–17]; however, the detailed mechanism for how to degrade RIP3 remains poorly understood. The E3 ligase CHIP can regulate cell necrosis through ubiquitylation- and lysosome-dependent RIP3 degradation [18]. The pellino E3 ubiquitin protein ligase 1 (PELI1) is shown to mediate K48-linked polyubiquitylation of RIP3 on lysine 363, leading to proteasomal degradation of RIP3 [19]. On the other hand, deubiquitinase A20 can deubiquitinate RIP3, thus hindering the formation of the RIP1-RIP3 necrosome and inhibiting the TNF-induced cell necrosis [20].

In this report, we discovered that the E3 ligase TRIM25 negatively regulates RIP3 stability through the ubiquitin-proteasome degradation pathway. TRIM25 directly binds to RIP3, promoting K48-linked polyubiquitination of RIP3 on residue K501, therefore inhibiting TNF-induced cell programmed necrosis. This provides a new homeostatic mechanism for post-translational modification of RIP3 to prevent aberrant cell death and inflammation.

## Results

### RIP3 directly interacts with TRIM25

**Fig. 1:**
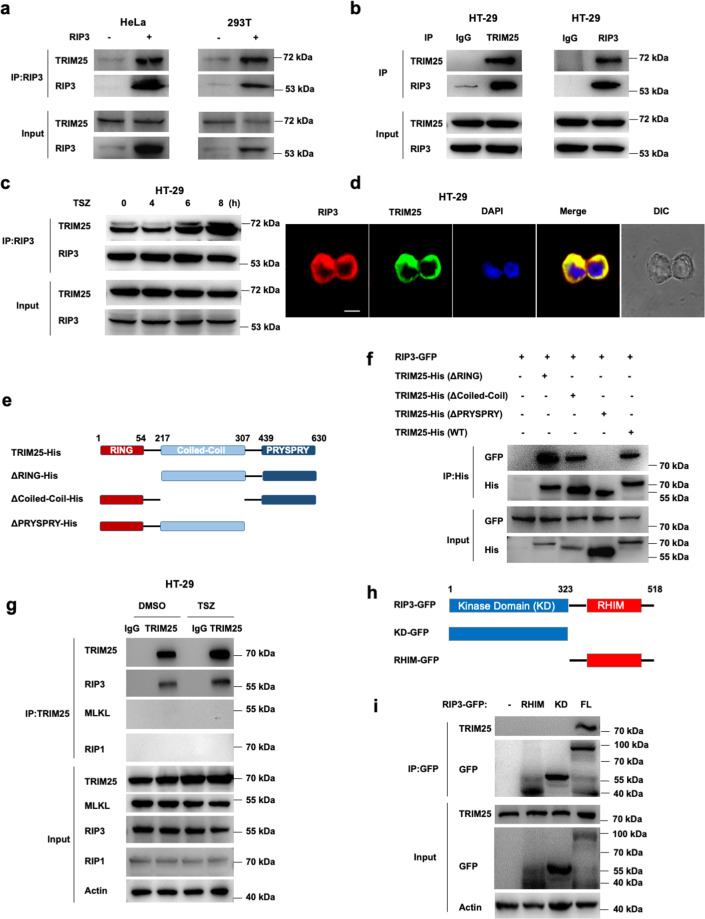
RIP3 interacts with TRIM25. **a** FLAG-tagged RIP3 was transfected into HeLa and HEK293T cells, followed by immunoprecipitation with the RIP3 antibody and immunoblotting analysis with TRIM25 and RIP3 antibodies, respectively. **b** Immunoprecipitation analysis with TRIM25 or RIP3 antibodies in HT-29 cells. **c** Co-immunoprecipitation analysis of RIP3 and TRIM25 by treatment with TNF/Z-VAD/Smac-mimetic (TSZ) stimuli for indicated hours in HT-29 cells. **d** RIP3 colocalized with TRIM25 in the cytoplasm of HT-29 cells. Endogenous RIP3 and TRIM25 were stained by anti-RIP3 (red) and anti-TRIM25 (green) antibodies, respectively. Scale bar, 10 µm. **e** Schematic of TRIM25 and different truncations. **f** His-tagged TRIM25 or its mutants and GFP-tagged RIP3 were co-transfected into HeLa cells. The cell lysates were immunoprecipitated with an anti-His antibody and then immunoblotted with the indicated antibodies. **g** HT-29 cells were primed with TSZ for 6 h, followed by immunoprecipitation with anti-TRIM25 antibody and probed with the indicated antibodies. **h** Schematic of RIP3 and different truncations. **i** GFP-tagged RIP3 or its mutants were transfected into HEK293T cells. The cell lysates were immunoprecipitated with an anti-GFP antibody and then immunoblotted with the indicated antibodies.

To reveal the potential regulators for RIP3 during cell necrosis, we performed a high-sensitivity mass spectrometry method by overexpressed human RIP3 in HeLa and HEK293T cells, followed by immunoprecipitation using an anti-RIP3 antibody. The results showed that the tripartite motif protein TRIM25 had a high sPSM score and could be the potential interacting protein of RIP3. To confirm the interaction between TRIM25 and RIP3, FLAG-tagged RIP3 was transfected into HEK239T and HeLa cells; it showed that RIP3 co-immunoprecipitated with endogenous TRIM25 (Fig. 1a). Further co-immunoprecipitation experiments in human colorectal adenocarcinoma HT-29 cells revealed that endogenous RIP3 interacted with TRIM25 (Fig. 1b). Furthermore, the interaction between endogenous TRIM25 and RIP3 was enhanced upon necroptotic stimuli (Fig. 1c). Also, endogenous RIP3 and TRIM25 colocalized in the cytoplasm of HT-29 cells (Fig. 1d).

TRIM25 contains a RING-finger domain (residues 1–54), a B-box/coiled-coil domain (residues 217–307), and a SPRY domain (residues 389–630) (Fig. 1e). To identify the key regions of TRIM25 for interaction with RIP3, co-immunoprecipitation experiments were performed for RIP3 with different TRIM25 mutants. As shown in Fig. 1f, the deletion of the RING-finger domain or the B-box/Coiled-coil domain dramatically decreased the binding ability with RIP3, whereas the deletion of the SPRY domain in TRIM25 lost the ability to interact with RIP3. Therefore, the SPRY domain is necessary for interaction with RIP3. We also found that TRIM25 specifically interacted with RIP3, but not RIP1 nor MLKL, the components of complex II (Fig. 1g). To explore the critical domain of RIP3 for the interaction between TRIM25 and RIP3, we performed co-IP experiments with truncated mutants of RIP3, including the kinase domain (KD) and the RHIM domain (Fig. 1h). The results showed that TRIM25 specifically interacted with full-length RIP3, but not the KD or the RHIM domain (Fig. 1i), implying that the stereochemical conformation of full-length RIP3 is needed for binding with TRIM25, similar to the reported cGAS-G3BP1 complex in the DNA sensing pathway [21]. Together, the above data indicated that RIP3 directly interacted with TRIM25 in vitro and in vivo.

### TRIM25 is required for the proteasomal degradation of RIP3

**Fig. 2:**
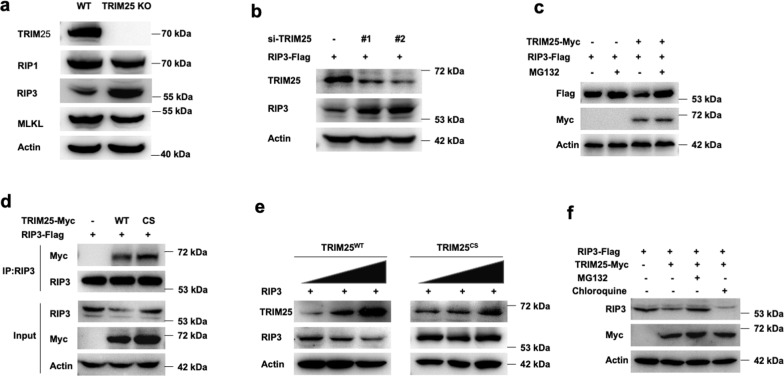
TRIM25 promotes the proteasomal degradation of RIP3. **a** Immunoblotting analysis of RIP1, RIP3, and MLKL in WT and TRIM25 KO HT-29 cells. **b** FLAG-tagged RIP3 was transfected into HeLa cells, then followed by the treatment with siRNAs of TRIM25. **c** FLAG-tagged RIP3 was co-transfected with or without Myc-tagged TRIM25 into HeLa cells, followed by the treatment by adding 10 uM MG132. **d** FLAG-tagged RIP3 was co-transfected with Myc-tagged TRIM25 or its mutant TRIM25^CS^ into HeLa cells, followed by the treatment with adding 10 uM MG132. **e** TRIM25 or TRIM25^CS^ were co-transfected with RIP3 in a dose-dependent manner in HeLa cells. **f** FLAG-tagged RIP3 was co-transfected with or without Myc-tagged TRIM25 into HeLa cells, followed by the treatment with MG132 (10 uM) or chloroquine (10 uM) for 4 h.

Previous studies showed that TRIM family proteins promoted the degradation of their substrates by the ubiquitin-proteasome pathway [22–24]. Therefore, we investigate whether TRIM25 could regulate the degradation process and affect the stability of RIP3. TRIM25 could decrease the protein level of RIP3, but not the upstream kinase RIP1 or the downstream pseudokinase MLKL in HT-29 cells (Fig. 2a). Consistently, knockdown of TRIM25 by small interfering RNAs (siRNA) significantly increased the protein level of RIP3 (Fig. 2b). Also, RIP3 protein levels were significantly enhanced in TRIM25 deficient cells when adding the proteasome inhibitor MG132 (Fig. 2c).

To elucidate whether TRIM25 acts as an E3 ligase to regulate RIP3, we generated an E3 ligase-defective TRIM25 mutant by mutating cysteines 50 and 53 to serine (C50S/C53S, denoted as TRIM25^CS^), which resulted in the loss of its E3 ubiquitin ligase activity [25]. The mutation did not change the binding ability as both TRIM25^WT^ and TRIM25^CS^ interacted with RIP3 in HeLa cells (Fig. 2d). Furthermore, an increase in TRIM25^WT^ expression significantly decreased RIP3 protein expression in a dose-dependent manner in HeLa cells, which indicated that TRIM25^WT^ could induce RIP3 degradation. However, TRIM25^CS^ did not change the protein level of RIP3, indicating that the E3 ligase activity of TRIM25 is required for RIP3 degradation (Fig. 2e). Also, TRIM25 significantly reduced RIP3 protein levels, which was restored by treating cells with the specific proteasome inhibitor MG132, but not by the lysosome inhibitor chloroquine (Fig. 2f). Taken together, these results indicated that TRIM25 promoted RIP3 degradation by the proteasomal pathway.

**Fig. 3:**
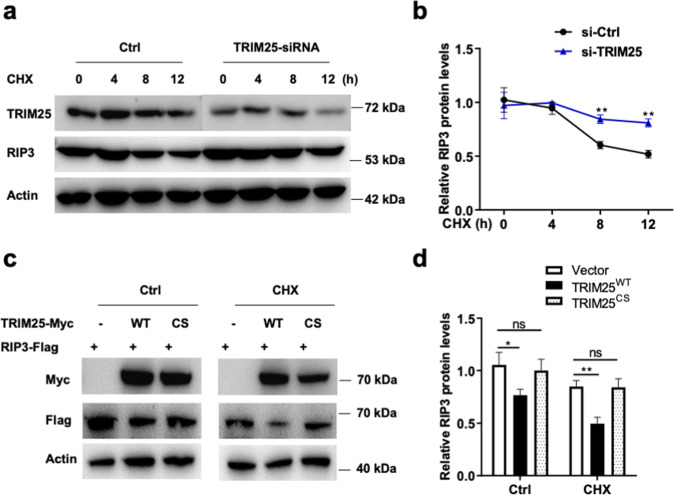
TRIM25 destabilizes RIP3 in cells. **a** RIP3-expressed HeLa cells were treated with TRIM25-siRNA in the presence of CHX for the indicated time. **b** Relative RIP3 protein levels were measured using ImageJ. **c**, **d** Myc-tagged TRIM25 or TRIM25^CS^ were co-transfected with FLAG-tagged RIP3 in the presence or absence of CHX for 12 h. The indicated proteins were measured by western blotting and quantitative using ImageJ (**d**).

Next, a cycloheximide (CHX) chase assay was used to investigate the effect of TRIM25 on RIP3 stability. As shown in Fig. 3a, b, TRIM25 knockdown inhibited RIP3 protein degradation in HeLa cells treated by CHX, suggesting that TRIM25 was a crucial regulator of RIP3 protein stability. Also, the half-life of RIP3 was significantly reduced by the overexpression of TRIM25^WT^ compared with control cells transfected with TRIM25^CS^ (Fig. 3c, d). These data suggested that TRIM25 stabilized RIP3.

### TRIM25 mediates the K48-linked polyubiquitination of RIP3

**Fig. 4:**
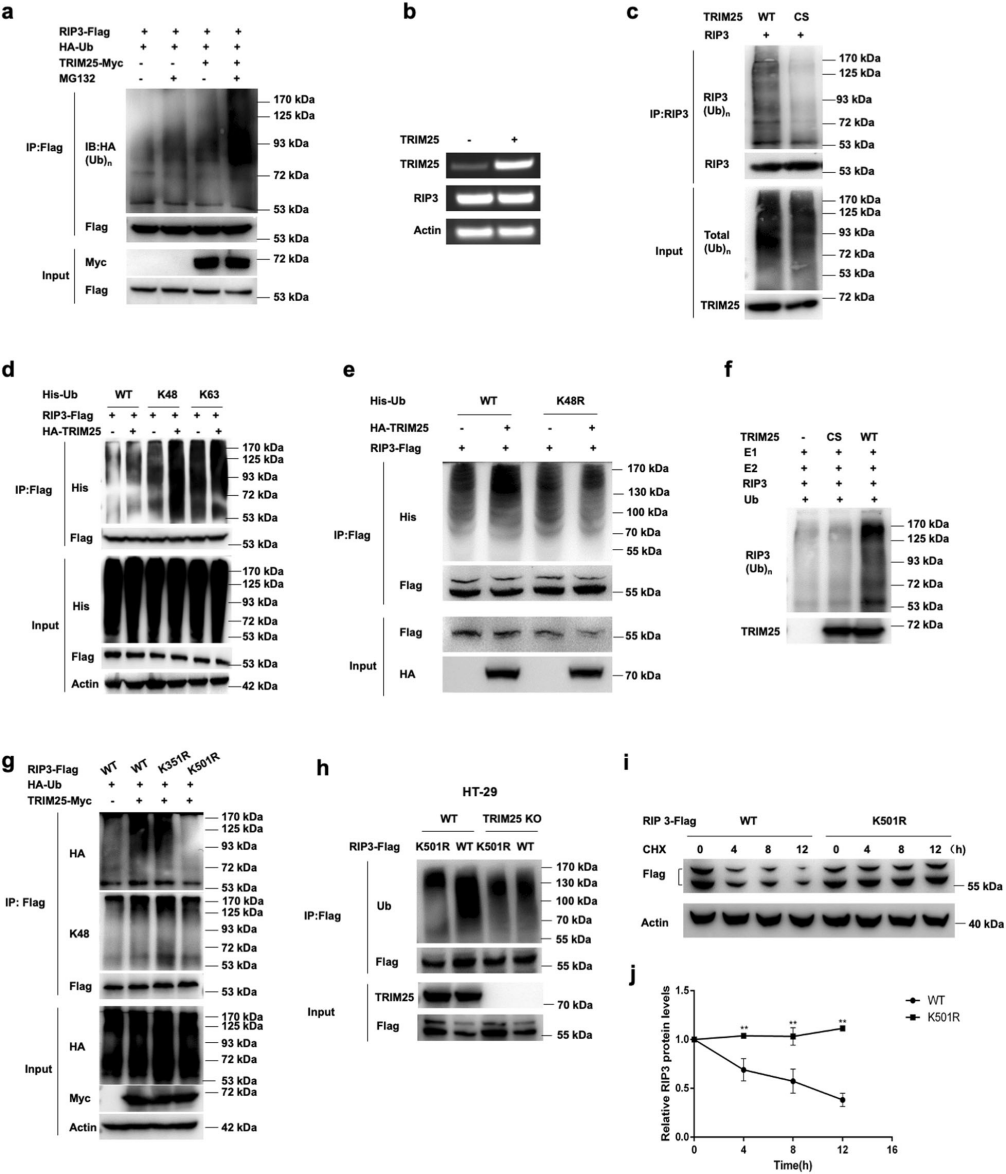
TRIM25 promotes the K48-linked polyubiquitination of RIP3. **a** HeLa cells were transfected with RIP3-FLAG and His-Ub or TRIM25-Myc, followed by immunoprecipitation with the anti-FLAG antibody and probed with the anti-Ub antibody. **b** RIP3 was transfected with or without TRIM25 into HeLa cells, followed by RT-PCR analysis. **c** RIP3 was co-transfected with TRIM25 or its mutant TRIM25^CS^, followed by immunoprecipitation with the anti-RIP3 antibody and probed with the anti-Ub antibody. **d** HeLa cells were transfected with FLAG-RIP3 and His-Ub (WT, k48 only or k63 only) with or without HA-TRIM25, followed by immunoprecipitation with the anti-FLAG antibody, and probed with the anti-His antibody. **e** HeLa cells were transfected with FLAG-RIP3 and His-Ub (WT or k48 mutant) with or without HA-TRIM25, followed by immunoprecipitation with the anti-FLAG antibody, and probed with the anti-His antibody. **f** In vitro ubiquitination assay for RIP3. Purified RIP3 was incubated with E1, E2, ubiquitin (Ub), TRIM25, or its mutant TRIM25^CS^ in the reaction buffer. **g** Co-immunoprecipitation analysis of the polyubiquitination of RIP3 and its mutants transfected with plasmids encoding TRIM25, FLAG-RIP3 (wild-type or its mutants), and His-Ub. **h** Co-immunoprecipitation analysis of the polyubiquitination of RIP3 and its mutant K501R in TRIM25 KO HT-29 cells. **i** HeLa cells were transfected with FLAG-RIP3 (WT or its mutant K501R) for 24 h, followed by treated with CHX for the indicated time. **j** Relative FLAG protein levels were measured using ImageJ.

As TRIM25 functions as an E3 ligase in antiviral immunity [26, 27], we asked whether TRIM25 could modify RIP3 by polyubiquitination. FLAG-tagged TRIM25 was co-transfected with HA-tagged ubiquitin and Myc-tagged RIP3 into HeLa cells. Overexpression of TRIM25 dramatically increased the ubiquitination of RIP3, especially in the presence of MG132 (Fig. 4a), whereas no apparent effects on RIP3 mRNA expression were detected (Fig. 4b). Notably, the RING domain mutant (TRIM25^cs^) lost the ability to mediate the polyubiquitination of RIP3 (Fig. 4c), indicating that TRIM25 could promote the ubiquitination of RIP3 via its N-terminal RING domain.

Protein ubiquitination plays vital roles in regulating diverse cellular processes, and different types of ubiquitination often lead to different biological events [28–30]. The two most abundant chain types are K48 and K63 linkages, which involve ubiquitin-proteasome degradation pathway and cell signaling pathways, respectively [31–33]. It has been reported that RIP3 could be ubiquitinated with both K48 and K63 linkages [18, 19, 33]. To identify the type of polyubiquitination, RIP3, together with His-Ub (WT, K48 only, K63 only) were transfected into HeLa cells in the presence or absence of TRIM25. The results showed that TRIM25 promoted the K48-linked polyubiquitination on RIP3, but no apparent effects on the K63-linked polyubiquitination (Fig. 4d). Furthermore, the parallel K48-mutated ubiquitin result showed no increment of RIP3 ubiquitination when TRIM25 was present (Fig. 4e). Next, we reconstituted the in vitro ubiquitination assay by adding the purified proteins TRIM25, human RIP3, E2 enzyme UbcH5A, E1 enzyme, and ubiquitin. The results showed that RIP3 robustly ubiquitinated RIP3 only when TRIM25 was present (Fig. 4f). To further investigate the potential ubiquitination sites of RIP3, two mutants K351R and K501R were introduced to perform the assay, which was based on the MS analysis and predicted by the ubiquitination sites prediction website (www.ubpred.org), respectively. It showed that the K501R but not K351R failed to be ubiquitinated by TRIM25, indicating K501 was the critical residue for ubiquitination (Fig. 4g). Furthermore, K501R did not impact the overall ubiquitination of RIP3 when TRIM25 was absent, indicating that K501 was a specific site for RIP3 ubiquitination (Fig. 4h). Consistent with these results, K501R mutant of RIP3 protein degradation was inhibited in HeLa cells treated by CHX, suggesting that K501 was a crucial residue of RIP3 protein stability (Fig. 4i, j). Taken together, TRIM25 induced the K48-linked polyubiquitination on the residue K501 of RIP3.

### TRIM25 is associated with TNF-induced necrosis

**Fig. 5:**
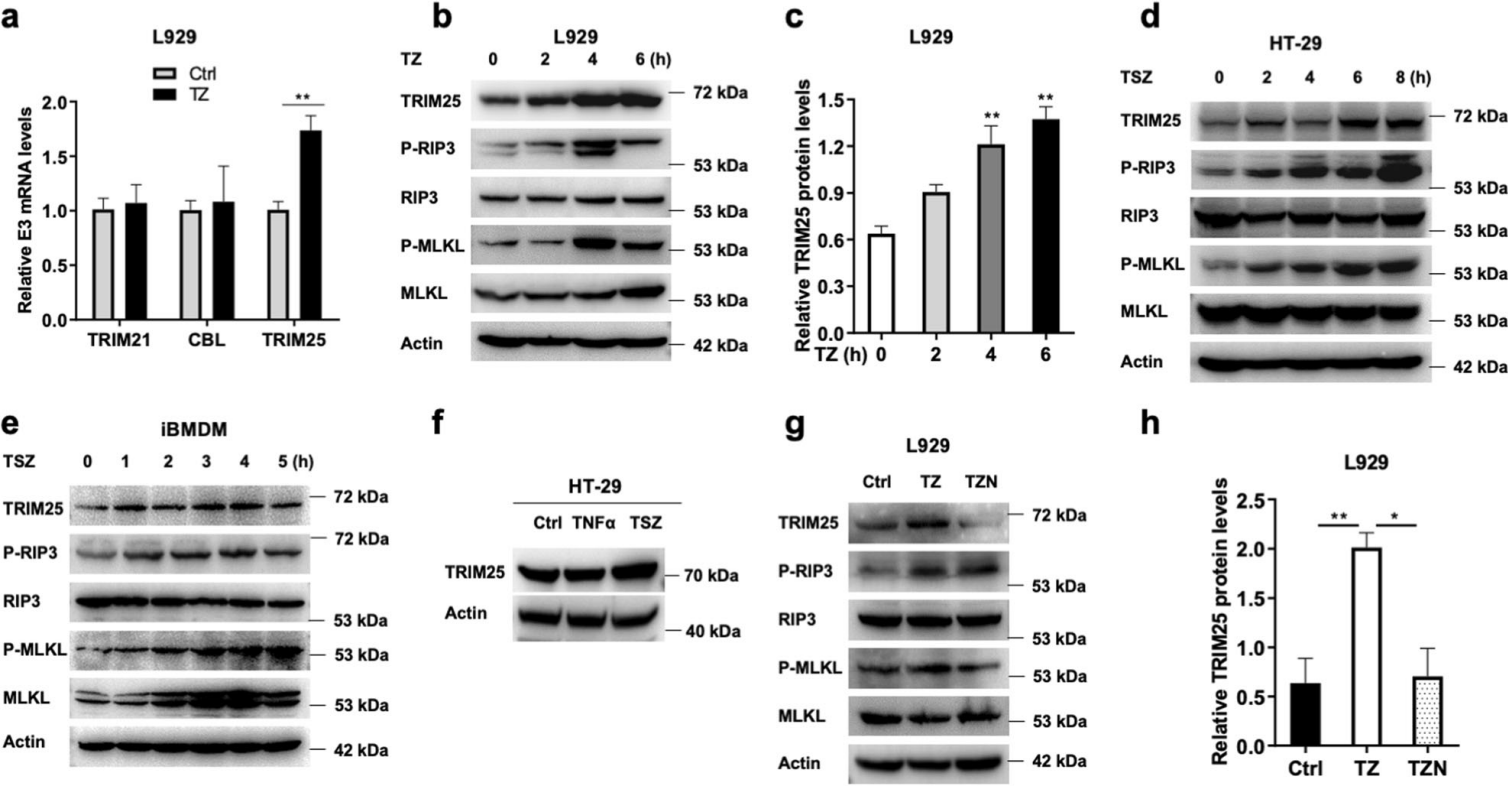
The necrotic stimuli induce TRIM25 expression. **a** qRT-PCR analysis of TRIM25 or other E3 ligases (TRIM21 and CBL) treated with TNF/Z-VAD (TZ) for 4 h in L929 cells. Immunoblotting analysis of TRIM25 treated with TZ for indicated hours in L929 cells (**b**), and quantitative by using ImageJ (**c**). **d** Immunoblotting analysis of TRIM25 treated with TSZ for indicated hours in HT-29 cells. **e** Immunoblotting analysis of TRIM25 treated with TSZ for indicated hours in iBMDM cells. **f** Immunoblotting analysis of TRIM25 treated with TSZ or TNF in HT-29 cells. Immunoblotting analysis of TRIM25 treated with TZ or TNF/Z-VAD/Nec-1 (TZN) for 4 h in L929 cells (**g**), and quantitative by using ImageJ (**h**).

To investigate the association of TRIM25 with cell necrosis, we examined the expression level of TRIM25 with the necrotic stimulation. After treatment with TNF plus Z-VAD (TZ) for 4 h, the mRNA level of TRIM25 increased significantly, whereas several other candidate E3 ligases TRIM21 and CBL did not change (Fig. 5a). Consistently, the protein level of TRIM25 increased with TZ stimulation in murine fibroblast L929 cells (Fig. 5b), which was significantly higher than that of no TZ stimulation in three independent experiments (Fig. 5c). Similar results were detected in HT-29 and iBMDM cells (Fig. 5d, e). The upregulation of TRIM25 was detected when HT-29 cells were treated with TSZ, but not with TNF only (Fig. 5f). Next, we used the small molecule Nec-1, a specific protein kinase inhibitor of RIP1 [34], to investigate the change of TRIM25 during cell programmed necrosis. As shown in Fig. 5g, h, TRIM25 increased significantly when treated with TZ, but decreased with the addition of Nec-1 in L929 cells. These data showed that TRIM25 was involved in TNF-induced cell necrosis.

### RIP3 undergoes robust ubiquitination during necrosis

**Fig. 6:**
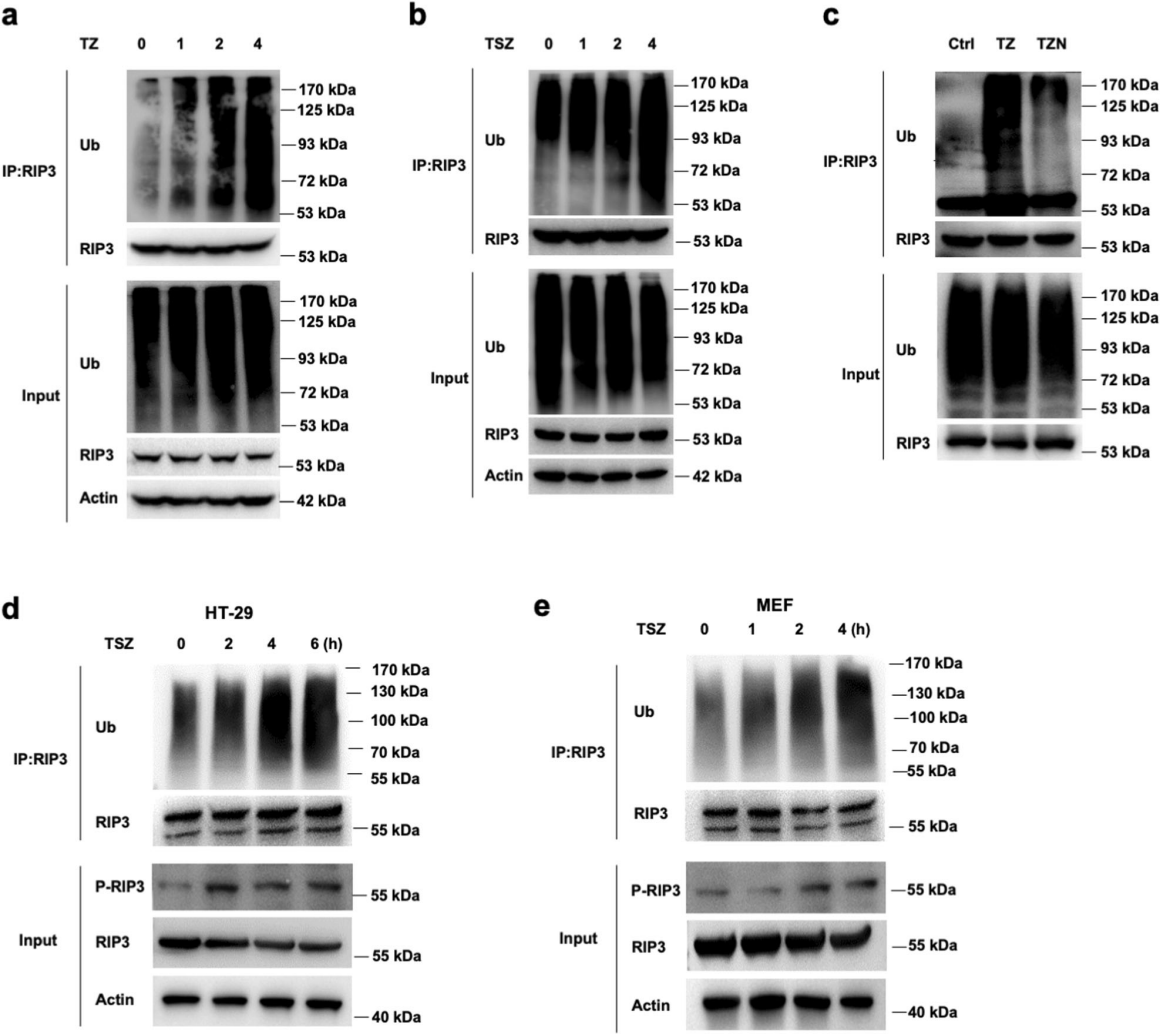
RIP3 is ubiquitinated during cell necrosis. **a** L929 cells were primed with TNF/Z-VAD (TZ) for different times, followed by immunoprecipitation with the anti-RIP3 antibody and probed with the anti-Ub antibody. **b** RIP3 transfected HeLa cells were primed with TSZ for different times, followed by immunoprecipitation with the anti-RIP3 antibody and probed with the anti-Ub antibody. **c** L929 cells were primed with TZ or TZN for 4 h, followed by immunoprecipitation with anti-RIP3 antibody and probed with the anti-Ub antibody. **d**, **e** HT-29 and MEF cells were primed with TSZ for indicated times, followed by immunoprecipitation in denaturing conditions with the anti-RIP3 antibody and probed with the anti-Ub antibody.

To investigate the ubiquitination status of RIP3 during necrosis, L929 cells were induced to undergo necrosis with TZ. Endogenous RIP3 from cultured L929 cells were immunoprecipitated to assess the ubiquitination of RIP3 by western blotting. The ubiquitination level of RIP3 was remarkably increased along with the time for TZ treatment when compared with the cells in the resting state (Fig. 6a). Consistently, transient-expressed RIP3 in HeLa cells exhibited robust ubiquitination when treated with TZ plus Smac mimetic (TSZ) (Fig. 6b). Furthermore, decreased ubiquitination of RIP3 was observed when cell necrosis was inhibited by the addition of Nec-1 (Fig. 6c). Also, similar results were found in HT-29 and MEF cells when RIP3 ubiquitination was detected in denaturing conditions (Fig. 6d, e). These results indicated that RIP3 could be ubiquitinated, and the ubiquitination level of RIP3 increased during TNF-induced cell necrosis.

### TRIM25 negatively regulates TNF-induced cell necrosis

**Fig. 7:**
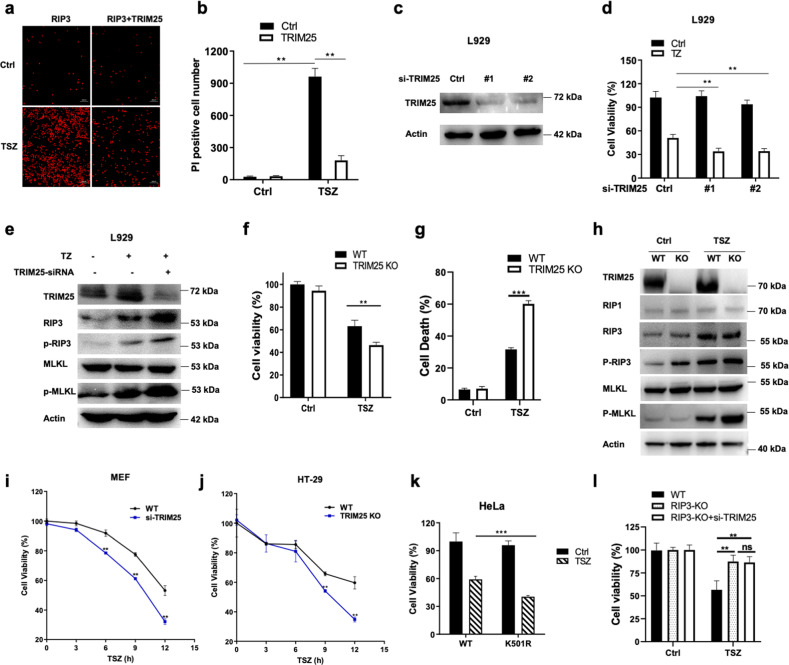
TRIM25 protects against TNF-induced cell necrosis. **a**, **b** HeLa cells were transfected with RIP3 and TRIM25 for 24 h, and then detected by PI staining. **c** Immunoblotting analysis of TRIM25 transfected with the corresponding siRNAs for 48 h in HeLa cells. **d** L929 cells were transfected with TRIM25 siRNA for 48 h and treated with TZ for another 4 h, and then detected by the MTT assay. **e** The control siRNA or TRIM25 siRNA were transfected into L929 cells for 48 h, and then treated with TZ for another 4 h. The corresponding proteins were detected by WB with different antibodies. **f** TRIM25 KO HT-29 cells or WT HT-29 cells were treated with TSZ for 6 h, and cell viability was detected by CCK-8 assay. **g** TRIM25 KO HT-29 cells or WT HT-29 cells were treated with TSZ for 6 h, and cell death was detected by LDH assay. **h** Immunoblotting analysis of the corresponding proteins with indicated antibodies in TRIM25 KO HT-29 cells or WT HT-29 cells after treated with TSZ for 6 h. **i**, **j** Time course effects of TRIM25 on protecting MEF cells or HT-29 cells against TNF-induced cell necrosis. **k** HeLa cells were transfected with K501R mutant of RIP3 or WT RIP3 for 24 h followed by TSZ stimulation for another 6 h, and then detected by MTT assay. **l** RIP3 KO or WT HT-29 cells were transfected with TRIM25 siRNA for 48 h and treated with TSZ for another 6 h. The cell viability was detected by the CCK-8 assay.

To further investigate the function of TRIM25 in TNF-induced cell necrosis, RIP3 alone or with TRIM25 were transfected into HeLa cells. The propidium iodide (PI) staining was used to assay the necrotic cells. The results showed that the TSZ-stimulated necrotic cells dramatically decreased when TRIM25 was co-transfected with RIP3 in HeLa cells (Fig. 7a, b). Furthermore, knockdown TRIM25 by the siRNA method showed that the number of viable cells was significantly reduced after TZ stimulation in the endogenous L929 cells (Fig. 7c, d). Also, the protein levels of phosphorylated RIP3 and phosphorylated MLKL dramatically increased after TZ stimulation in L929 cells (Fig. 7e). Similar results were found in TRIM25 KO HT-29 cells, evidenced by the decreased cell viability and the increased cell necrosis upon necroptotic stimulation (Figs. 7f, g). Meanwhile, the necroptotic markers phosphorylated RIP3 and phosphorylated MLKL significantly enhanced upon TSZ stimulation in TRIM25 KO HT-29 cells compared to WT HT-29 cells (Fig. 7h). To further investigate the protective effect of TRIM25, time-course experiments were conducted in mouse embryonic fibroblast (MEF) cells and HT-29 cells. As expected, the results showed that TRIM25 had a protective effect against necroptotic cell death upon TSZ stimulation along time in MEF and HT-29 cells (Fig. 7i, j). As TRIM25 induced the K48-linked polyubiquitylation on the residue K501 of RIP3, the effect of mutant K501R of RIP3 on cell death was examined. As shown in Fig. 7k, the cell viability was significantly decreased upon TSZ stimulation in RIP3 K501R mutant HeLa cells compared with WT RIP3 expressed HeLa cells, indicating that K501 was a crucial residue of RIP3 for cell necrosis (Fig. 7k). Also, we found that TRIM25 knockdown did not affect on TNF-induced cell death in RIP3 KO HT-29 cells (Fig. 7l). These results demonstrated that TRIM25 inhibited the process of TNF-induced cell necrosis and had a protective effect against cell death.

## Discussion

Cell programmed necrosis functions as an alternative pathway to apoptosis in various cell activities [35, 36]. RIP3, RIP1, and MLKL form a large complex that triggered cell necrosis upon TNF signaling. RIP3 is reported to undergo various post-translational modifications, including phosphorylation, acetylation, and ubiquitination, during cell necrosis signaling pathways [37–39]. Two E3 ligases, CHIP and PELI1, are reported to be in charge of the degradation of RIP3 [18, 19]. CHIP can control cell necroptosis by ubiquitylation- and lysosome-dependent degradation of RIP3 [18], whereas PELI1 targets kinase active RIP3 by the ubiquitination-dependent proteasomal degradation [19]. The current study identified TRIM25 as a RIP3 interacting protein and a potential negative regulator in TNF-induced cell necrosis. Tripartite motif (TRIM)-containing proteins control essential cellular processes such as innate immunity, antiviral response, intracellular signaling, cell apoptosis, autophagy, and carcinogenesis [24, 40–43]. Previous reports showed that TRIM25 mediates the K63-linked polyubiquitination on RIG-I and IFIH1, functioning in innate immune defense against viruses [26, 27]. On the other way, NLRP12 interacts with TRIM25 to prevent TRIM25-mediated polyubiquitination and activation of RIG-I, therefore dampening RIG-I-mediated antiviral immune signaling [44]. Also, TRIM25 can promote the ISGylation of the adapter protein 14-3-3 sigma (SFN) [45], as well as mediating the ubiquitination and subsequent proteasomal degradation of ZFHX3 [46]. In contrast, TRIM25 induced a K48-linked ubiquitin moiety to the K501 residue of RIP3, promoting RIP3 degradation and inhibited TNF-induced cell necrosis (Fig. 4).

TRIM25 contains multiple domains (Fig. 1e). The E3 ligase activity of TRIM25 was dependent on its RING domain (Fig. 4), similar to that in other substrates [26, 27]. Also, TRIM25 interacts with influenza A virus NS1 protein via its coiled-coil domain; this interaction in turn inhibits TRIM25 polymerization and the ubiquitination of RIG-I [47]. During cell necrosis, the interaction between TRIM25 and RIP3 was mediated by the SPRY domain (Fig. 1f), similar manner as that between TRIM25 and RIG-I. In addition, knock-down TRIM25 significantly inhibited the polyubiquitination of RIP3, and promoted TNF-induced cell necrosis in multiple cell lines (Figs. 5 and 7). As RIP3 is involved in various inflammatory diseases, the intervention of the degradation process induced by TRIM25 might be a potential therapeutic target in the future.

## Materials and methods

### Plasmid construction and transfection

Human TRIM25 was constructed by PCR-based amplification of cDNA from HEK293T cells, and then subcloned into the pcDNA3-HA and pEGFP-N1 vectors, respectively. FLAG-tagged RIP3 and mCherry-tagged RIP3 were gifts kindly provided by Dr. Francis Chan (Duke University, USA). Truncated mutants and point mutants of TRIM25 and RIP3 were generated using the KOD-Plus-Mutagenesis kit. Expression vectors for His-Ub (wild type, K48 only, and K63 only) were gifts from Dr. Ronggui Hu (Chinese Academy of Sciences, Shanghai, China). Expression vectors for His-Ub (wild type and K48 mutant) were gifts from Dr. Chengjiang Gao (Shandong University, Shandong, China). All constructs were confirmed by DNA sequencing. Plasmids were transiently transfected into HEK293T cells or HeLa cells with Lipofectamine 2000 (Invitrogen) according to the manufacturer’s instructions.

### siRNA and reverse transcription-PCR (RT-PCR)

siRNAs for TRIM25 were #1 (5′- GCAAAUGUACCCAGCACAATT-3′) and #2 (5′- GGAUGUGAGAUCCAAGCAATT-3′) from GenePharma (Shanghai, China). The siRNA duplexes were transfected into L929 cells using Lipofectamine RNAi MAX (Invitrogen). The total RNA of indicated cell lines was extracted with TRIzol reagent according to the manufacturer’s instructions (Invitrogen). Specific primers used for RT-PCR assays were 5′-ACTACAATACCGCCCACAAC-3′, 5′-CATCCACAAGACAATCACCC-3′ for TRIM25; 5′-GGACCTCAAGCCCTCTAACA-3′, 5′-ACCTCGGAGACAGCAGCATC-3′ for RIP3; 5′-AAGAAGAACCTGCCCGATGA-3′, 5′-CCGAATGGTGTAGCCTGTAT-3′ for MLKL; 5′-TGTTACCAACTGGGACGACA-3′, 5′-CTGGGTCATCTTTTCACGGT-3′ for β-actin. The gene expression levels were normalized to those of β-actin.

### Cell culture

HEK293T, HeLa, L929, HT-29, iBMDM, and MEF cells were grown in high-glucose-containing DMEM (Hyclone) supplemented with 10% FBS (Hyclone). TRIM25 KO HT-29 cells were generated using CRISPR/Cas9 technology. RIP3 KO HT-29 cells were kindly provided by Dr. Huayi Wang (ShanghaiTech University, Shanghai, China). All cells were grown at 37 °C under a humidified 5% CO_2_ atmosphere (Thermo Fisher). L929 cells were stimulated with 10 ng/mL TNF (Abcam) and 5 μM Z-VAD-FMK (Abcam) to induce cell necrosis, while other cells were induced necrosis with 100 nM Smac mimetic (MCE) addition.

### Immunoprecipitation and immunoblotting analysis

For immunoprecipitation (IP), whole-cell extracts were lysed in an IP buffer composed of 1% (vol/vol) NP-40, 150 mM NaCl, 1 mM EDTA, 50 mM Tris-HCl (pH 7.5) and the complete protease inhibitor cocktail (Roche). Cell lysates were collected and incubated with 1 μg of the corresponding antibodies together with protein G Plus-Agarose Immunoprecipitation reagent (Santa Cruz). After overnight incubation at 4 °C, agaroses were washed five times and boiled with the IP buffer for SDS-PAGE. For immunoblotting analysis, cells were lysed with a lysis buffer (150 mM NaCl, 1 mM EDTA, 1% Triton X-100 and 50 mM Tris-HCl, pH 7.5) supplemented with the protease inhibitor cocktail (Roche), and were incubated with the following antibodies: anti-FLAG (Proteintech, 20543-1-AP), anti-HA (Proteintech, 51064-2-AP), anti-His (Proteintech, 66005-1-lg), anti-RIP1 (Santa Cruz, sc-133102), anti-RIP3 (Santa Cruz, sc-374639), anti-RIP1(Santa Cruz, sc-133102), anti-Myc (Proteintech, 67447-1-Ig), anti-pRIP3 (Abcam, ab205421), anti-mouse pMLKL (Abcam, ab196436), anti-human pMLKL (Abcam, ab206336), anti-MLKL (Abcam, ab243142), anti-Actin (Abcam, ab8226), and anti-TRIM25 (Abcam, ab167154).

### Recombinant protein production and in vitro ubiquitination assay

The full-length RIP3, TRIM25, E1, E2 (UbcH5A), and ubiquitin were subcloned into the pSMT3 vector with an N-terminal His-SUMO tag, respectively. All these proteins were expressed in *E. coli* BL21 (DE3) cells and then purified by Ni-affinity chromatography. The His-SUMO tag was cleaved with the Ulp1 enzyme followed by the second Ni-affinity chromatography purification. Fractions throughout the column were collected and concentrated and then were purified with the gel-filtration chromatography (Superdex G75 10/300, GE). For in vitro ubiquitination assay, 5 μg of purified RIP3 was mixed in a total volume of 50 μL containing 1 μg E1, 1 μg E2, 5 μg ubiquitin, 5 mM ATP, and 5 mM MgCl_2_ in the presence or absence of the TRIM25 protein. After incubation at 37 °C for 1 h, reactions were stopped by adding a 5× loading buffer and then analyzed by western blotting using the anti-RIP3 antibody.

### Immunofluorescence staining and confocal analysis

For immunofluorescence analysis, cells were washed in PBS three times and then fixed in 4% paraformaldehyde for 15 min at room temperature. The fixed cells were permeabilized with a buffer containing 0.1% Triton X-100, and then incubated in a blocking solution including 5% BSA in PBS for 30 min. Primary antibodies were diluted in PBS at 1:100 and incubated overnight at 4°C, followed by secondary antibodies diluted in PBS at 1:1000 and incubated for 1 h at room temperature away from light. Nuclei were stained with DAPI (P36931, Invitrogen). The cells staining with PI were observed with 535 nm excitation and 615 nm emission filters. Cells were observed with confocal laser microscopy (LSM710, Carl Zeiss, Germany).

### Statistical analysis

Each experiment was performed at least three times. All experiment data were analyzed using GraphPad Prism 6.0 (GraphPad software Inc. USA), and presented as mean values ± SEM. Statistical analysis was performed using a Student’s *t* test or One-way analysis of variance. (*: *p* < 0.05; **: *p* < 0.01).
